# Impact of catheter ablation and subsequent recurrence of atrial fibrillation on glucose status in patients undergoing continuous glucose monitoring

**DOI:** 10.1038/s41598-023-31139-0

**Published:** 2023-03-15

**Authors:** Masako Baba, Kentaro Yoshida, Akihiko Nogami, Yuichi Hanaki, Yasuaki Tsumagari, Masayuki Hattori, Hideyuki Hasebe, Akito Shikama, Hitoshi Iwasaki, Noriyuki Takeyasu, Masaki Ieda

**Affiliations:** 1 Department of Cardiology, Ibaraki Prefectural Central Hospital, Kasama, Japan; 2 Department of Cardiology, Institute of Medicine, University of Tsukuba, 1-1-1 Tennodai, Tsukuba, 305-8575 Japan; 3 Department of Endocrinology and Metabolism, Ibaraki Prefectural Central Hospital, Kasama, Japan; 4 Department of Endocrinology and Metabolism, Institute of Medicine, University of Tsukuba, Tsukuba, Japan

**Keywords:** Cardiology, Endocrinology

## Abstract

Although glucose metabolism and atrial fibrillation (AF) have complex interrelationships, the impact of catheter ablation of AF on glucose status has not been well evaluated. Continuous glucose monitoring (CGM) with a FreeStyle Libre Pro (Abbott) was performed for 48 h pre-procedure, during the procedure, and for 72 h post-procedure in 58 non-diabetes mellitus (DM) patients with symptomatic AF and 20 patients with supraventricular or ventricular arrhythmias as a control group. All ablation procedures including pulmonary vein isolation were performed successfully. Glucose levels during procedures consistently increased in the AF and control groups (83.1 ± 16.1 to 110.0 ± 20.5 mg/dL and 83.3 ± 14.7 to 98.6 ± 16.3 mg/dL, respectively, P < 0.001 for both), and Δ glucose levels (max minus min/procedure) were greater in the AF group than control group (P < 0.001). There was a trend toward higher mean glucose levels at 72 h after the procedures compared with those before the procedures in both the AF and control groups (from 103.4 ± 15.6 to 106.1 ± 13.0 mg/dL, P = 0.063 and from 100.2 ± 17.1 to 102.9 ± 16.9 mg/dL, P = 0.052). An acute increase in glucose level at the time of early AF recurrence (N = 9, 15.5%) could be detected by simultaneous CGM and ECG monitoring (89.7 ± 18.0 to 108.3 ± 30.5 mg/dL, P = 0.001). In conclusion, although AF ablation caused a statistically significant increase in the glucose levels during the procedures, it did not result in a pathologically significant change after ablation in non-DM patients. Simultaneous post-procedure CGM and ECG monitoring alerted us to possible acute increases in glucose levels at the onset of AF recurrence.

## Introduction

Catheter ablation for atrial fibrillation (AF) has emerged as a well-established treatment option^1,2^. However, long-term maintenance of sinus rhythm is commonly difficult especially in patients with comorbidities such as metabolic syndrome. Although glucose metabolism disorder is one of the risk factors for occurrence of AF in the general population and for recurrence of AF after catheter ablation^3,4^, and substantial metabolic alternation in AF pathophysiology has been reported^5–7^, the impact of catheter ablation on the procedural glucose status has not been well evaluated. Myocardial damage by ablation lesion sets causes local or systemic inflammation^8–10^ and may modify autonomic nervous activities^11,12^. Non-cardiac factors, such as use of anesthetics, beta-antagonists, and heparin, ablation-related pain, and psychological stress, may also modify metabolism. These factors can potentially contribute to changes in glucose status and arrhythmia recurrence soon after AF ablation.

A comprehensive record of the changes in glucose levels can now be obtained using continuous glucose monitoring (CGM). CGM helps us to know how glucose status changes over time, and specific patterns of glycemic responses may reflect underlying physiology such as physiological and psychological stress, inflammation, and autonomic remodeling^13^. Also, simultaneous glucose and electrocardiographic (ECG) monitoring after ablation may document an acute change in the glucose level at the time of early recurrence of AF. These observations may give us insights into the multifactorial mechanisms underlying the clinically significant relation between glucose metabolism disorder and AF.

## Methods

### Study design and subjects

This study prospectively enrolled 58 patients with symptomatic AF who underwent initial catheter ablation at Ibaraki Prefectural Central Hospital between April 2019 and February 2022. A separate group of 20 patients who underwent catheter ablation of arrhythmias other than AF served as a control group. Their arrhythmias comprised Wolff-Parkinson-White syndrome in 4, atrioventricular nodal reentrant tachycardia in 4, cavo-tricuspid isthmus-dependent atrial flutter in 3, and ventricular premature complexes (VPCs) in 9 patients. No patients in the control group had a history of AF. Excluded patients included those who were previously diagnosed as having diabetes mellitus (DM) by elevated HbA1c level > 6.5% or were taking anti-diabetic medications, those with a history of ablation, and those with symptomatic heart failure. All anti-arrhythmic drugs and beta-blockers were discontinued at least five half-lives before ablation.

### Continuous glucose monitoring

Professional sensors for CGM (FreeStyle Libre Pro; Abbott GmbH & Co. KG) were used. The accuracy of the FreeStyle Libre Pro was reported to show an absolute difference between CGM and the plasma glucose level of 11.4% in patients with diabetes^14^ and 10.5% in individuals with normoglycemia^15^. The sensor, which consists of a thin needle placed in the subcutaneous tissue, measures the interstitial glucose level every 15 min for up to 14 days. Its small size allows free movement and performance of normal daily activity and causes less stress among the patients.

The sensor was inserted in the upper arm for at least 7 days in all patients. The CGM data were analyzed for 3 periods: for 48 h before ablation, during ablation, and for 72 h after ablation. The data recorded in the first 24 h were excluded from the analysis due to the need for stabilization between the sensor and the interstitial fluid after insertion of the device, and the 24 h before ablation were also excluded from the analysis because transesophageal echocardiography to exclude thrombus in the left atrial appendage was performed in this period. The period during ablation was defined as that from the femoral puncture to removal of the sheath. The period after ablation was defined as the 72 h beginning immediately after sheath removal. In addition, if AF recurred within 72 h after ablation, the glucose data from 15 min before the onset of AF to 90 min after its onset were analyzed.

### Catheter ablation

All patients fasted for at least 6 h before the procedure and were infused with lactated Ringer’s solution from 2 h before. Transesophageal echocardiography and cardiac computed tomography were also performed prior to the procedure to exclude left atrial thrombus and to reconstruct the left atrial anatomy. The electrophysiological study and catheter ablation were performed under conscious sedation with dexmedetomidine and fentanyl. Unfractionated heparin was administered to maintain an activated clotting time between 300 and 350 s. Surface electrocardiograms and intracardiac electrograms were continuously monitored and stored on an EP-WorkMate recording system (Abbott, Saint Paul, MN). Blood pressure was monitored continuously through a femoral arterial access line. A 6F 20-pole dual-site mapping catheter (BeeAT; Japan Lifeline Co., Ltd., Tokyo, Japan) was inserted through the subclavian vein and positioned in the coronary sinus, right atrium, and superior vena cava throughout the procedure. An intracardiac echocardiography catheter (AcuNav, Biosense Webster, Diamond Bar, CA) was advanced into the right atrium via the femoral approach to guide the transseptal puncture. Two long sheaths (SL0; AF Division, Abbott) were advanced into the left atrium. Pulmonary vein isolation was performed by point-by-point radiofrequency ablation with 3D electroanatomic mapping (CARTO 3 system, Biosense Webster). The radiofrequency current was delivered via a ThermoCool (Biosense Webster) catheter with power up to 35 W. The endpoint was the achievement of bidirectional conduction block between the left atrium and the pulmonary veins. A positive ganglionated plexus (GP) response during ablation was defined as transient ventricular asystole and atrioventricular block^16^. When non-pulmonary vein ectopies were reproducibly observed with and without continuous infusion of isoproterenol (1–4 µg/min), they were targeted for ablation. The patients with a clinical history of typical atrial flutter or induced flutter during the procedure underwent cavotricuspid isthmus ablation.

### Arrhythmias and biological assessments after ablation

After the procedure, patients remained hospitalized under continuous ECG monitoring for at least 3 days in the AF group and for at least one day in the control group to detect early recurrences of the targeted arrhythmias. For assessment of an acute inflammatory process after ablation, body temperature was measured every 6 h, and blood samples were taken to measure C-reactive protein (CRP) and troponin-T levels at one day after ablation. AF recurrence was defined as the appearance of AF or atrial tachycardia lasting > 30 s.

### Statistical analysis

Variables with a normal distribution are presented as the mean ± SD, and those with a skewed distribution are presented as the median (interquartile range). Categorical variables are expressed as numbers and percentages. For continuous variables, an unpaired Student *t*-test or the Mann–Whitney U test was used to test for differences between the two groups, whereas for categorical variables, the Fisher’s exact test was used. The changes in the glucose level during the procedure and at the onset of AF recurrence were analyzed by general linear model repeated measure. Logistic regression analysis was performed to determine independent predictors of early (within 72 h after ablation) recurrence of AF. Differences between pre- and post-ablation glucose levels were tested using a paired Student *t*-test. Statistical significance was set at P < 0.05. All statistical analyses were performed using JMP version 12.0 (SAS Institute Inc., Cary, NC).

### Ethics approval and consent to participate

The study protocol was approved by the institutional review board of Ibaraki Prefectural Central Hospital, Kasama, Japan (IRB No. R1-4,) and complied with the principles of the Declaration of Helsinki. Written informed consent was obtained from all patients.

## Results

### Patient characteristics

Baseline characteristics of the patients are presented in Table 1. The mean age of the patients was 61.4 years, and 32.1% were female. The age in the AF group was significantly higher and the body surface area (BSA) was greater than those in the control group. The baseline HbA1c was similar between the AF and control groups (5.9 ± 0.5% vs 5.9 ± 0.5%, P = 0.764). In the AF group, 50% of patients had paroxysmal AF.

**Table 1:** Baseline clinical characteristics.

Characteristic	All patients (n = 78)	AF group (n = 58)	Control group (n = 20)	P
Female, n (%)	25 (32.1)	14 (24.1)	11 (55.0)	0.011
Age (years)	61.4 ± 11.3	63.1 ± 10.2	56.6 ± 13.2	0.026
Body mass index (kg/m^2^)	24.9 ± 3.5	25.3 ± 3.6	23.6 ± 2.9	0.055
Body surface area (m^2^)	1.8 ± 0.2	1.8 ± 0.2	1.7 ± 0.2	0.005
CHADS2 score	1.0 (0–1.0)	1.0 (0–1.0)	0.5 (0–1.0)	0.354
Paroxysmal AF, n (%)	N/A	29 (50.0)	N/A	N/A
Coexisting conditions				
Sick sinus syndrome, n (%)	11 (14.1)	11 (19.0)	0 (0)	0.036
Hypertension, n (%)	35 (44.9)	27 (46.6)	8 (40.0)	0.612
Stroke, n (%)	8 (10.3)	6 (10.3)	2 (10.0)	0.965
Sleep apnea syndrome, n (%)	8 (10.3)	8 (13.8)	0 (0)	0.080
Structural heart disease, n (%)	9 (11.5)	6 (10.3)	3 (15.0)	0.574
Ischemic heart disease, n (%)	5 (6.4)	5 (8.6)	0 (0)	0.175
Echocardiographic findings				
Ejection fraction (%)	63.0 ± 9.0	63.3 ± 8.7	61.8 ± 10.4	0.573
LAVi (mL/m^2^)	34.5 ± 13.2	36.1 ± 13.6	27.9 ± 9.4	0.035
Laboratory data				
HbA1c (%)	5.9 ± 0.5	5.9 ± 0.5	5.9 ± 0.5	0.764
eGFR (mL/min/1.73 m^**2**^)	70.2 ± 15.3	70.2 ± 15.2	70.4 ± 15.9	0.957
BNP (pg/mL)	39.0 (13.0–80.0)	41.5 (15.0–82.3)	26.0 (10.5–66.9)	0.331
CGM parameters before ablation (48 h)				
Mean glucose (mg/dL)	102.5 ± 15.9	103.4 ± 15.6	100.2 ± 17.1	0.453

*AF* atrial fibrillation, *LAVi* left atrial volume index, *eGFR* estimated glomerular filtration rate, *BNP* brain natriuretic peptide, *CGM* continuous glucose monitoring.

### Procedural characteristics

The total procedure time and radiofrequency ablation time were significantly longer in the AF group than those in the control group (255.0 ± 47.7 vs 166.7 ± 60.5 min, P < 0.001 and 57.2 [52.1–69.5] vs 9.1 [3.4–12.4] min, P < 0.001, respectively). There was no significant difference in the CRP level after ablation, but the troponin-T level and body temperature were higher in the AF group than those in the control group (troponin-T: 1.1 [0.9–1.3] vs 0.2 [0–0.3] ng/L, P < 0.001; body temperature: 37.3 ± 0.4 vs 36.9 ± 0.5 °C, P < 0.001, respectively) (Table 2). GP responses were observed in 21 patients (36.2%) in the AF group.

**Table 2:** Procedural characteristics.

Characteristic	All patients (n = 78)	AF group (n = 58)	Control group (n = 20)	P
Procedure time (min)	233.2 ± 63.5	255.0 ± 47.7	166.7 ± 60.5	< 0.001
Ablation time (min)	53.6 (30.9–63.1)	57.2 (52.1–69.5)	9.1 (3.4–12.4)	< 0.001
Inflammatory parameters after ablation				
C-reactive protein (mg/dL)	0.5 (0.3–0.7)	0.5 (0.4–0.7)	0.3 (0.1–0.7)	0.087
Troponin-T (ng/L)	1.0 (0.6–1.2)	1.1 (0.9–1.3)	0.2 (0–0.3)	< 0.001
Body temperature (°C)	37.2 ± 0.4	37.3 ± 0.4	36.9 ± 0.5	< 0.001

*AF* atrial fibrillation.

### Glucose profiles at baseline (48 h before ablation)

The mean glucose level at baseline (48 h before ablation) was comparable between the AF and control groups (103.4 ± 15.6 vs 100.2 ± 17.1 mg/dL, P = 0.453) (Table 1). In the AF group, the mean glucose level was similar between the patients with paroxysmal AF and those with persistent AF (103.1 ± 11.9 vs 103.7 ± 18.7 mg/dL, P = 0.905). Also, there was no difference in the mean glucose level between patients with sinus rhythm during the pre-ablation period (N = 35, 60.3%) and those with an AF rhythm (N = 23, 39.7%) (102.9 ± 11.6 vs 104.0 ± 20.3 mg/dL, P = 0.806).

### Glucose status during the procedures

**Figure 1:**
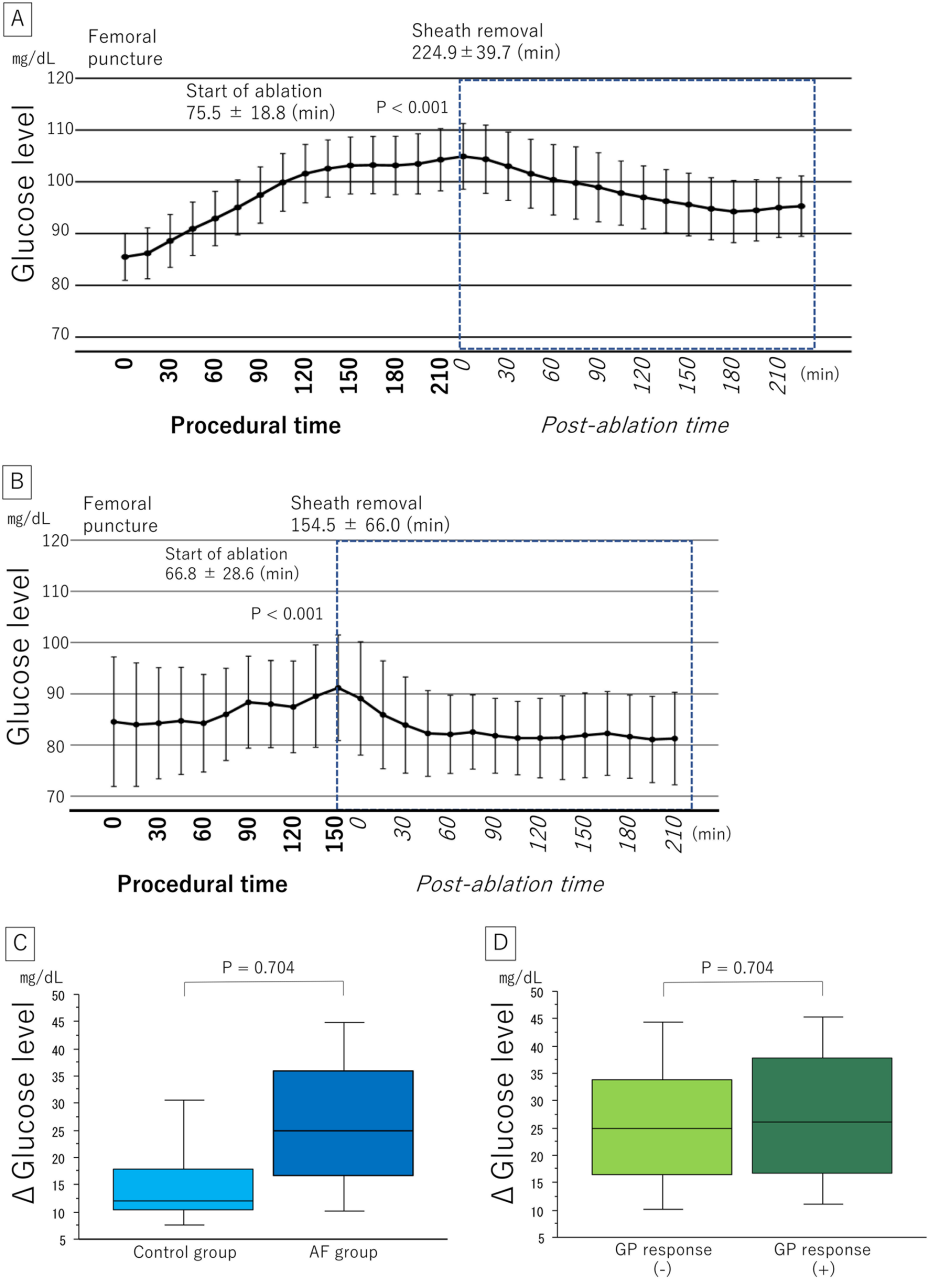
(**A**,**B**) Interstitial glucose levels during and after the procedure in the AF group and the control group. (**C**) The Δ glucose level (maximum minus minimal) during the procedure in the AF and control groups. Time zero was defined as the time of femoral puncture. The mean time from femoral puncture to the start of ablation was 75.5 ± 18.8 min in the AF group and 66.8 ± 28.6 min in the control group. The mean time from femoral puncture to sheath removal was 224.9 ± 39.7 min in the AF group and 154.5 ± 66.0 min in the control group. (**D**) The Δ glucose level in patients with and without GP responses. *AF* atrial fibrillation, *GP* ganglionated plexus.

The fluctuations in the glucose level during the procedure in the AF and control groups are presented in Fig. 1A,B, respectively. The glucose level increased consistently in both the AF and control groups (from 83.1 ± 16.1 to 110.0 ± 20.5 mg/dL and 83.3 ± 14.7 to 98.6 ± 16.3 mg/dL, P < 0.001, respectively). The Δ glucose level (maximum minus minimum) during the procedure in the AF group was higher than that in the control group (25.5 [17.0–36.0] vs 12.0 [10.5–18.0] mg/dL, P < 0.001) (Fig. 1C). In the AF group, there was no difference in the Δ glucose level during the procedure between patients with and without GP responses (26.0 [16.8–37.8] vs 25.0 [16.5–33.8] mg/dL, P = 0.704) (Fig. 1D).

### Glucose profiles 72 h after ablation

**Figure 2:**
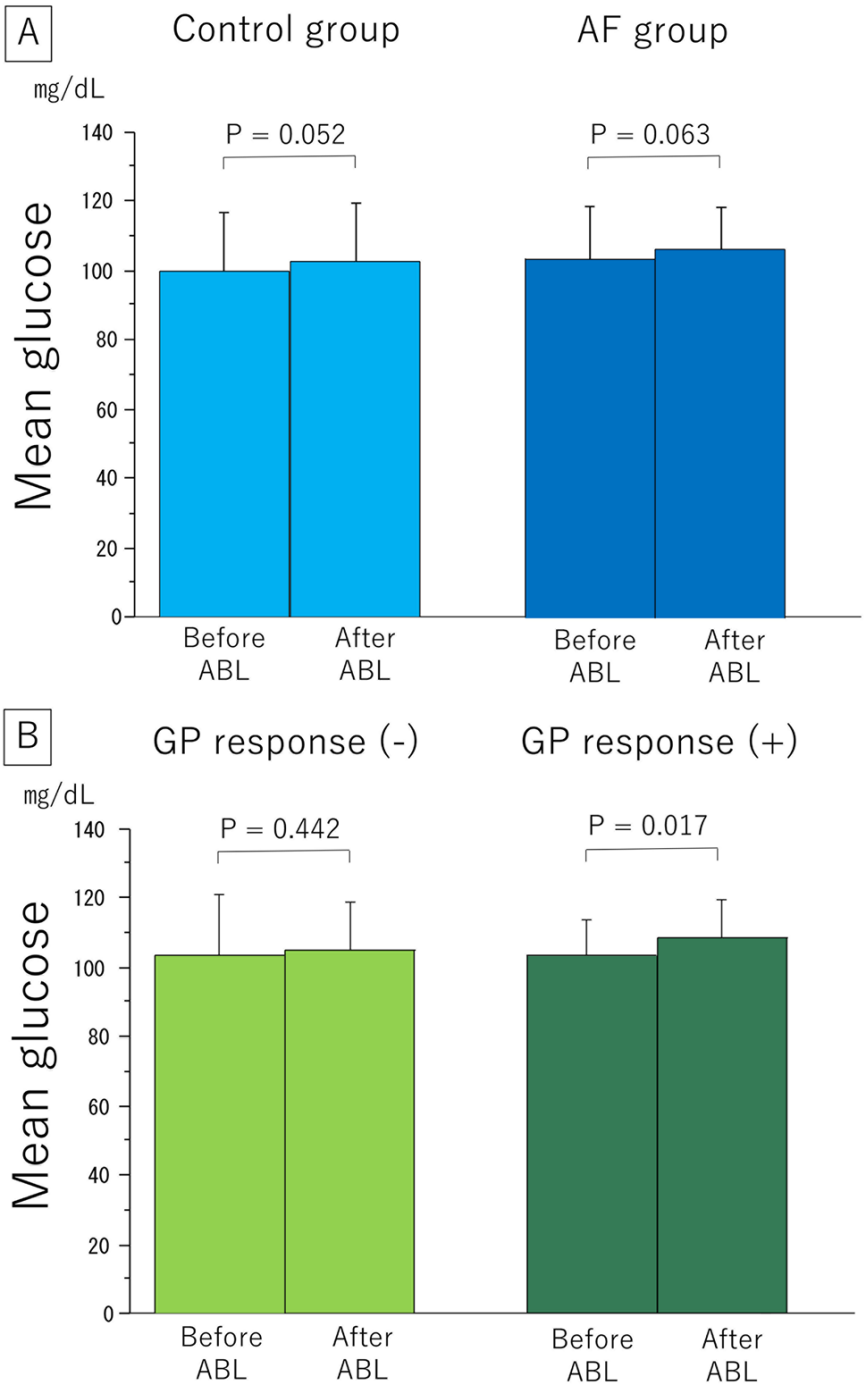
(**A**) The mean absolute change in the glucose level before and after ablation. (**B**) In the AF group, the mean glucose level was separately assessed in patients with and without GP responses. *ABL* ablation, *AF* atrial fibrillation, *GP* ganglionated plexus.

There was a trend toward higher mean glucose levels at 72 h after the procedures than those before the procedures in both the AF and control groups (from 103.4 ± 15.6 to 106.1 ± 13.0 mg/dL, P = 0.063 and from 100.2 ± 17.1 to 102.9 ± 16.9 mg/dL, P = 0.052) (Fig. 2A). In the AF group, there was no increase in the glucose level after ablation in patients without a GP response (103.4 ± 17.8 to 104.9 ± 13.9 mg/dL, P = 0.442), although patients with a GP response had a significant increase (103.3 ± 10.7 to 108.3 ± 11.1 mg/dL, P = 0.017) (Fig. 2B).

### Characteristics of early recurrence of AF

There was no recurrence of arrhythmia in the control group. Among the 58 patients who underwent AF ablation, 9 patients (15.5%) experienced AF recurrence within 72 h after ablation. The median time to AF recurrence was 31.75 h (range 9.75–63 h). The characteristics of the patients with and without AF recurrences are shown in Table 3.

**Table 3:** Baseline clinical characteristics in patients with and without AF recurrence within 72 h after ablation.

Characteristic	AF occurring within 72 h after ablation		P
	(+) n = 9	(−) n = 49	
Female, n (%)	3 (33.3)	11 (22.4)	0.483
Age (years)	62.8 ± 12.0	63.1 ± 10.0	0.940
Body mass index (kg/m^2^)	22.4 ± 2.0	25.9 ± 3.6	0.007
Body surface area (m^2^)	1.6 ± 0.2	1.8 ± 0.2	0.012
CHADS2 score	0 (0–2.0)	1.0 (0–1.0)	0.513
Paroxysmal AF, n (%)	3 (33.3)	26 (53.1)	0.277
Duration of AF (months)	48 (17–96)	25 (8–51)	0.308
Coexisting conditions			
Sick sinus syndrome, n (%)	4 (44.4)	7 (14.3)	0.034
Hypertension, n (%)	2 (22.2)	25 (51.0)	0.111
Stroke, n (%)	1 (11.1)	5 (10.2)	0.935
Sleep apnea syndrome, n (%)	1 (11.1)	7 (14.3)	0.800
Structural heart disease, n (%)	2 (22.2)	4 (8.2)	0.203
Ischemic heart disease, n (%)	0 (0)	5 (10.2)	0.316
Echocardiographic findings			
Ejection fraction (%)	65.4 ± 2.9	62.9 ± 9.4	0.429
LAVi (mL/m^2^)	39.0 ± 13.4	35.6 ± 13.6	0.491
Laboratory data			
HbA1c (%)	5.9 ± 0.5	5.8 ± 0.5	0.830
eGFR (mL/min/1.73 m^2^)	64.6 ± 16.0	71.2 ± 15.0	0.235
BNP (pg/mL)	82.3 (25.8–103.3)	39.9 (14.5–73.3)	0.128
CGM parameter: before ablation period (48 h)			
Mean glucose (mg/dL)	97.3 ± 14.4	104.4 ± 15.7	0.235

*AF* atrial fibrillation, *LAVi* left atrial volume index, *eGFR* estimated glomerular filtration rate, *BNP* brain natriuretic peptide, *CGM* continuous glucose monitoring.

There was no difference in the mean glucose level before ablation between patients with and without AF recurrence. The body mass index (BMI) and BSA were smaller in the patients with AF recurrence than in those without (22.4 ± 2.0 vs 25.9 ± 3.6 kg/m^2^, P = 0.007 and 1.6 ± 0.2 vs 1.8 ± 0.2 m^2^, P = 0.012, respectively). Sick sinus syndrome was more common in the patients with AF recurrence (44.4% vs 14.3%, P = 0.034). No significant differences were noted in the distribution of paroxysmal versus persistent AF or in echocardiographic parameters between the two groups. The ablation time in patients with AF recurrence was significantly longer than that in those without recurrence (73.2 ± 16.0 vs 58.7 ± 15.8 min, P = 0.015). Troponin-T level and body temperature after ablation were higher in the patients with AF recurrence than in those without (1.5 [1.0–1.7] vs 1.1 [0.8–1.2] ng/L, P = 0.065 and 37.6 ± 0.4 °C vs 37.3 ± 0.3 °C, P = 0.020, respectively) (Table 4). Multivariate analysis showed no independent predictors for early AF recurrence (Supplemental Table).

**Table 4:** Procedural characteristics in patients with and without AF recurrence within 72 h after ablation.

Characteristic	AF occurring within 72 h after ablation		P
	(+) n = 9	(−) n = 49	
Procedure time (min)	277.2 ± 35.3	250.7 ± 48.9	0.127
Ablation time (min)	73.2 ± 16.0	58.7 ± 15.8	0.015
CTI block, n (%)	2 (22.2)	9 (18.4)	0.786
SVCI, n (%)	2 (22.2)	14 (28.6)	0.695
Focal atrial, n (%)	4 (44.8)	9 (18.4)	0.085
GP response during procedure, n (%)	5 (55.6)	16 (32.7)	0.189
Inflammatory parameters after AF ablation			
C-reactive protein (mg/dL)	0.4 (0.2–0.9)	0.5 (0.4–0.7)	0.361
Troponin-T (ng/L)	1.5 (1.0–1.7)	1.1 (0.8–1.2)	0.065
Body temperature (°C)	37.6 ± 0.4	37.3 ± 0.3	0.020

*AF* atrial fibrillation, *CTI* cavotricuspid isthmus, *SVCI* superior vena cava isolation, *GP* ganglionated plexus.

### Glucose status at the onset of early recurrence

**Figure 3:**
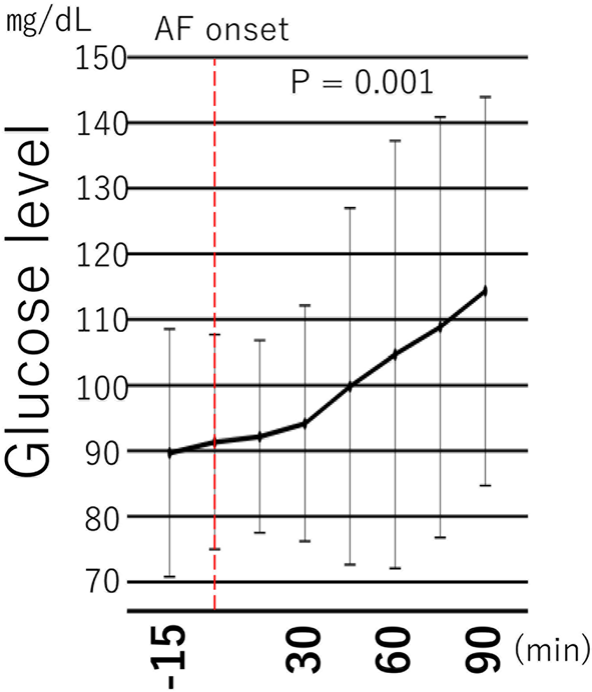
Interstitial glucose level immediately after the onset of early recurrence of atrial fibrillation (AF).

To clarify whether higher glucose level is a cause or effect of AF recurrence, we focused on the acute change in the glucose level at the time of AF recurrence. Nine patients experienced early AF recurrence within 72 h after ablation. To eliminate the influence of diet in the analysis of glycemic variability at the time of AF recurrence, we excluded 3 patients in whom AF recurrence occurred after a meal. In the remaining 6 patients, the onset of AF recurrence occurred up to 90 min before the meal. In this subgroup in which the effect of dietary glycemic change could be excluded, the glucose level increased significantly from 15 min before AF onset to 90 min after AF onset (from 89.7 ± 18.0 to 108.3 ± 30.5 mg/dL, P = 0.001) (Fig. 3), whereas there was no significant increase in the glucose level during fasting in patients without early recurrence of AF (N = 49) (data not shown).

## Discussion

To our best knowledge, this is the first study to evaluate both the periprocedural glucose status of non-DM patients undergoing AF ablation and the glucose status at the onset of early AF recurrence.

The potentially important and novel findings of our study are as follows:

1. The glucose levels gradually and consistently increased during the ablation procedure in both the AF and control groups, but the extent of the increase was greater in the AF group than that in the control group. No adverse events such hypo- or hyperglycemia occurred during the procedures.
2. There was a trend toward higher mean glucose levels at 72 h after the procedures than that before the procedures in both the AF and control groups. In the AF group, the presence of GP responses was associated with the increase in the glucose level after ablation.
3. During the 72-h period after ablation, AF recurred in 9 (16%) of the 58 patients. Acute inflammation such as pericarditis characterized by an increase in fever and troponin-T elevation may be associated with these recurrences. Notably, an immediate increase in the glucose level was observed at the time of early AF recurrence in these patients.

The glucose level during the procedures was increased despite fasting in both groups. AF ablation affected the glucose level more significantly than did other procedures, paroxysmal supraventricular tachycardia, and VPCs. One possible mechanism is the increase in the sympathetic activities due to mental and physical stress particularly because of the conscious sedation used in this series. The longer procedure time, longer ablation time, pain typically occurring during left atrial posterior wall ablation, and ganglionated plexus ablation may possibly explain the greater increase in sympathetic activities in the AF group. Another mechanism might be a weakness in glucose tolerance that was originally present in the patients with AF. It is widely known that even patients without previous evidence of DM can have transient hyperglycemia due to external factors and environments, namely “stress hyperglycemia”^17^. The development of stress hyperglycemia is caused by a highly complex interplay of counter-regulatory hormones such as catecholamines, growth hormone, cortisol, and cytokines^18,19^. Although patients with DM were excluded from this study, subclinical impairment of glucose tolerance could be present in the AF patients. The AF group was older than the control group, and impaired glucose tolerance is also one of the common risk factors for AF development in the general population^20^. It is well known that systemic inflammation causes an elevation in blood glucose^21,22^. However, neither a high fever of > 38 °C nor a clinically significant elevation in the CRP level was observed in this patient series, and it is unclear whether local (atrial) inflammation associated with pericarditis would lead to elevation of the glucose level.

Diabetes leads to increased morbidity and length of stay of surgical patients. One of the reasons is hypo- and hyperglycemia. Hypoglycemia sometimes manifests as drowsiness, which may be wrongly attributed to sedation. NHS Diabetes guidelines for the perioperative management of the adult patient with diabetes recommend glucose monitoring for patients undergoing general anesthesia if the patient receives insulin and the procedure is longer than 1–2 h^23,24^. However, there are no data, no recommendations, and no guidelines regarding glucose monitoring during catheter ablation despite the long procedure time of over 2 h and the use of conscious sedation or general anesthesia. Although hypo- and hyperglycemias could occur in theory during ablation of AF, the present results first provide electrophysiologists with actual evidence of little risk from these factors and little need for routine glucose measurements during the procedures in non-diabetes patients. However, because only non-diabetes patients were included in the present study, the presence or absence of hypo- and hyper-glycemia in diabetes-patients should be of great interest to electrophysiologists to improve safety of the procedures. In this regard, the present study may provide future direction for further studies investigating glucose status during ablation procedures.

In this study, there was a trend toward higher mean glucose levels at 72 h after the procedures than that before the procedures in both the AF and control groups. Although this was a small change (% increase of ~ 3%), and at least in non-DM patients, the effect of ablation may be transient and limited to the period during the procedure, this study implied that autonomic change by GP ablation may affect glucose metabolism after ablation in patients with AF.

One more important result was the acute increase in the glucose level following AF onset. There is no data, to our knowledge, regarding the acute effect of AF onset on the glucose level in humans because continuous monitoring of both ECG and glucose level has been difficult to perform. The patients with immediate AF recurrence were characterized by small BSA and BMI, longer ablation and radiofrequency times, and a greater increase in body temperature and CRP and troponin-T levels after ablation, suggesting the higher likelihood of acute inflammation probably associated with pericarditis^8–10^. Symptoms such as palpitations and chest discomfort, anxiety, and hemodynamic change in addition to inflammation and autonomic imbalance could also be reasons for the glucose elevation. These results may provide an important message to physicians treating patients with DM and AF in that unexplained hyperglycemia might occur when AF events are frequent especially when the AF is asymptomatic. The simultaneous application of ECG monitoring and CGM over a longer-term period is currently difficult, but it may provide further insights into glucose metabolism as a cause and effect of AF, i.e., a vicious circle, in future studies.

Several limitations should be acknowledged. First, this is a single-center study, and the number of patients is relatively small. Second, calorie intake at home before ablation could not completely controlled. However, eating habits were reviewed when instruction on CGM was provided in the outpatient clinic, and calorie intake during hospital admission was consistent (1800 kcal). Third, because the present study is the first, to our knowledge, to evaluate the glucose level during ablation procedures and, therefore, focused on patients who are estimated to have higher risk for hypo- and hyperglycemia, i.e., patients with AF, glucose levels during other types of procedures such as percutaneous coronary artery intervention and pacemaker implantation, should also be of great interest to cardiologists. These additional evaluations may contribute to further clarification of the mechanisms involved in the interplay between AF and DM. Finally, patients with DM were excluded from the study because this was a first study to evaluate the pure effect of AF ablation on glucose metabolism. Further studies including those on patients with DM may provide insights into the pathological interplay between glucose metabolism, the development of AF, and AF ablation.

In conclusion, AF ablation resulted in a significant increase in the glucose level during the procedures, but it did not cause a pathologically significant change early (within 72 h) after ablation in non-DM patients. Simultaneous CGM and ECG monitoring post-procedure alerted us to the acute increase in the glucose levels at the onset of AF recurrence, which suggested multifactorial contributions to glucose metabolism such as inflammation, autonomic imbalance, mental stress, and hemodynamic impairment.

## Supplementary Information

Supplementary Information.

## Data Availability

The datasets used and/or analyzed during the current study are available from the corresponding author on reasonable request.

## Abbreviations

AF: Atrial fibrillation
BMI: Body mass index
BSA: Body surface area
CGM: Continuous glucose monitoring
CRP: C-reactive protein
DM: Diabetes mellitus
ECG: Electrocardiographic
GP: Ganglionated plexus
VPCs: Ventricular premature complexes
