# Supplementary material for: Impact of catheter ablation and subsequent recurrence of atrial fibrillation on glucose status in patients undergoing continuous glucose monitoring
Source: Sci Rep. 2023 Mar 15;13:4299. doi: 10.1038/s41598-023-31139-0 (PMC10017667; doi:10.1038/s41598-023-31139-0)
Supplement: Supplementary file 1 — Supplementary Information. [file 41598_2023_31139_MOESM1_ESM.docx]

| **Supplemental table**  Logistic regression analysis for AF recurrence within 72 h after ablation | | | | | |  |
| --- | --- | --- | --- | --- | --- | --- |
| **Variable** | **Univariate** | |  | **Multivariate** | | |
|  | **OR (95% CI)** | **P** |  | **OR (95% CI)** | **P** | |
| Sick sinus syndrome | 4.800 (1.030-22.374) | 0.0458 |  | 1.916 (0.208-17.681) | 0.5662 | |
| Body mass index | 0.600 (0.404-0.890) | 0.0110 |  | 0.670 (0.447-1.003) | 0.0515 | |
| Ablation time | 1.001 (1.000-1.002) | 0.0248 |  | 1.001 (1.000-1.003) | 0.1008 | |
| Troponin-T | 7.105 (1.272-39.672) | 0.0254 |  | 0.320 (0.012-8.377) | 0.4936 | |
| Body temperature | 10.184 (1.255-82.633) | 0.0298 |  | 15.617 (0.629-388.053) | 0.0936 | |
| OR: odds ratio, CI: confidence interval. | | | | | | |
